# Supplementary material for: Indoor air pollution prevention practices and associated factors among household mothers in Olenchiti town, Oromia, Ethiopia
Source: PLoS One. 2024 Jan 19;19(1):e0296706. doi: 10.1371/journal.pone.0296706 (PMC10798486; doi:10.1371/journal.pone.0296706)
Supplement: S1 File — (PDF) [file pone.0296706.s001.pdf]

Information sheet and consent form

Introduction and Consent form that certifies the respondent's agreement before answering the questions

Dear mother:

Hello. My name is _____________ and we are working on indoor air pollution prevention practices and associated factors among household mothers in Olenchiti town, Oromia, Ethiopia. The objective of this study is to assess the level of indoor air pollution prevention practices and associated factors among households in Olenchiti town, East Shewa zone, Oromia, Ethiopia, 2022. This information will help the town to plan health services. Therefore, your honest and genuine participation in responding to the interview is highly appreciated and helpful to attain the objective of the study. The interview usually takes around 20 minutes to complete.

Confidentiality: As part of the study we would like to ask some questions about the indoor air pollution (IAP) status of your household. To ensure confidentiality, your name will not be written on this form. Besides, the interview will be made in a place where it is conducive to you, in your compound. Whatever information you provide will be kept strictly confidential, and will not be shared with anyone other than members of our study team.

Withdrawal without prejudice: Participation in this study is voluntary, and if we should come to any question you don't want to answer, just let me know and I will go on to the next question, or you can stop the interview at any time. However, we hope you will participate in the study since your views are important.

Risk and Discomforts: There are no risks or discomforts that are anticipated from your participation in the study.

Benefits: The anticipated benefit of participation is the opportunity to discuss indoor air pollution prevention practices of households.

Cost and /or payment to the subject for participation in research: There is no cost for participation in the research.

Agreement: At this time, do you want to ask me anything about the study?

May I begin the interview now?

1. yes---- 2.No----

If your answer is yes you can start answering the questions. But if your answer is no, Thank you, your will stop here.

Signature of interviewer: ----------------------Date: ----------------

For any questions contact the principal investigator Worku Dugassa Mobile-0926180577

English version questionnaires

Part I – Socio-demographic Characteristics questions

These questions are asked **to the mother of the household only**. The interviewers will read each question carefully to them and write or circle their responses in the given spaces.

NO	Questions	Responses
1.	Housing number	__________________
2.	Family size	________________
3.	Do you have under-five children	1. Yes 2. No
4.	If yes, the total number of under-five children	_________________
5.	Your Sex	1. Male 2.Female
6.	Your age in years	______ Years
7.	Your Ethnicity	1. Oromo 2. Amhara<br>3. Gurage 4. Walayita<br>5.Other(Specify)_________
8.	Your Religion	1. Muslim 2. Orthodox<br>3. Protestant 4. Catholic<br>5. Other (Specify)__________
9.	What is your educational status?	1. Unable to read and write<br>2. Read and write<br>3. Grade 1-8 4. Grade 9-12<br>5. Diploma and above
10.	What is your occupation?	1. Housewife 2. Merchant<br>3. Governmental employee<br>4. Daily laborer 5. Private employee<br>6. Farmer 7. Others --------------
11.	Monthly average income	_________________birr/month

Part II- Housing-related questions (please observe if necessary)

No	Questions	Responses
12.	Do you have a separate kitchen from the living room (observe)	1. Yes 2. No
13.	Does the cooking room (kitchen) have a window? (observe)	1. Yes 2. No
14.	If yes, how many windows? (observe)	____________
15.	Does the cooking room (kitchen) have a chimney? (observe)	1. Yes 2. No
16.	What type of stove is used in the cooking room (kitchen)? (observe)	1. Modern stove<br>2. Traditional stove
17.	If modern stoves, list out.	1. Mixadi<br>2. electric stove<br>3. Midija

		4. others ________
18.	What is the roof construction of the cooking room (kitchen)? (observe)	1. Corrugated iron sheet<br>2. Traditional thatch roof<br>3. Others _____
19.	Does the cooking room (kitchen) have been cleaned (no dust on the floor, wall, and ceiling)?	1. Yes 2. No

Part III: Household's Sources of energy questions

No	Questions	Responses
20.	Which source of energy is used by your households?	1. Yes 2. No
	Electricity	1. Yes 2. No
	Wood (crop waste)	1. Yes 2. No
	Charcoal	1. Yes 2. No
	Animal dung	1. Yes 2. No
	Kerosene	1. Yes 2. No
	Mixed sources of energy (electricity & wood products)	1. Yes 2. No

Part IV: Household's behavioral factors questions

No	Questions	Responses
21.	Do you have a cigarette smoker in your family member?	1. Yes 2. No
22.	If yes, did you smoke indoor house?	1. Yes 2. No
23.	Do you open windows or doors while cooking?	1. Yes 2. No
24.	Do you use charcoal after complete combustion outside the house	1. Yes 2. No
25.	Do you separate under five children from the kitchen while cooking?	1. Yes 2. No
26.	Do you cook for more than 1hr in the kitchen without going out?	1. Yes 2. No
27.	Do you get any training related to IAP during the last 6 months from the health or energy bureau?	1. Yes 2. No
28.	Do you get any follow-up related to the prevention of IAP during the last 6 months from the health or energy bureau?	1. Yes 2. No
29.	Did your household member have any diseases during the last two weeks?	1. Yes 2. No
30.	If yes, specify the diseases	________________
**Part V: Knowledge of the effects of IAP**		
31.	Do you think that IAP causes the following problems?	
	Respiratory diseases	1. Yes 2. No
	Lung cancer	1. Yes 2. No
	Low birth weight	1. Yes 2. No
	Impaired cognitive development	1. Yes 2. No

	Cataract	1. Yes 2. No
	Global climate change	1. Yes 2. No

Part VI: Mothers' IAP prevention practices

No	Questions	Responses
32.	Use of separate kitchen	1. Yes 2. No
33.	Use of modern stove	1. Yes 2. No
34.	Opening window or door while cooking	1. Yes 2. No
35.	Separating under-five children from the kitchen while cooking	1. Yes 2. No
36.	Outside complete combustion of charcoal	1. Yes 2. No
37.	Use of clean energy source (electricity)	1. Yes 2. No
