# Supplementary material for: Indoor air pollution prevention practices and associated factors among household mothers in Olenchiti town, Oromia, Ethiopia
Source: PLoS One. 2024 Jan 19;19(1):e0296706. doi: 10.1371/journal.pone.0296706 (PMC10798486; doi:10.1371/journal.pone.0296706)
Supplement: Attachment — Submitted filename: PONE-D-23-21169_R1.pdf [file pone.0296706.s004.pdf]

PLOS ONE

Indoor air pollution prevention practices and associated factors among household mothers in Olenchiti town, Oromia, Ethiopia

--Manuscript Draft--

**Manuscript Number:**	PONE-D-23-21169R1
**Article Type:**	Research Article
**Full Title:**	Indoor air pollution prevention practices and associated factors among household mothers in Olenchiti town, Oromia, Ethiopia
**Short Title:**	Indoor air pollution prevention practices and associated factors among household mothers in Oromia, Ethiopia
**Corresponding Author:**	Worku Dugassa Girsha, MPH<br>Adama General Hospital and Medical College<br>Adama, Oromia ETHIOPIA
**Keywords:**	indoor air pollution; prevention practices; associated factors; Olanchity town; Ethiopia
**Abstract:**	Introduction<br><br>Most households in low- and middle-income countries still cook using solid fuels such as wood, crop wastes, charcoal, coal, dung and kerosene in poorly ventilated dwellings and inefficient stoves. Indoor air pollution cause a range of health problems, like pneumonia, lung cancer, stillbirth, low birth weight, and impaired cognitive development and cataracts. Nevertheless, a few evidences are available in the Africa, including Ethiopia. Therefore, this study was aimed to assess the level of indoor air pollution prevention practices and associated factors among household mothers in Olenchiti town, Oromia, Ethiopia.<br><br>Methods<br><br>A community based cross sectional study was conducted. Four hundred twenty mothers were randomly selected by systematic random sampling. Data was collected through interview and observation checklist. The collected data entered into Epi-Info version 7.2.5 cleaned, edited and then exported to SPSS version 23 for analysis. Descriptive statistics was used to describe findings. Binary logistic regression was computed to analyze the effect of each variable on the outcome variable. Adjusted odds ratio with 95% confidence interval and P -value <0.05 was used as cut-off points to declare significance in the final model.<br><br>Results<br><br>The overall good practices of mothers towards indoor air pollution was 45.0%. Mothers who had under five children (AOR =0.49, 95%CI (0.31-0.76), mothers in grade 9 – 12 educational level (AOR=0.51, 95%CI (0.28 - 0.92)), merchant mothers (AOR = 0.37, 95%CI (0.14-0.96) were significantly associated with indoor air pollution prevention practices.<br><br>Conclusion<br><br>The overall good practices of mothers towards indoor air pollution was low compared to different findings. The factors significantly associated with indoor air pollution prevention practices in the final model were under five children, educational status and occupation. Therefore, indoor air pollution topics should be included in high school curriculum.
**Order of Authors:**	Worku Dugassa Girsha, MPH
	Alem Deksisa Abebe, MPH
	Ephrem Mannekulih Habtewold, MSc

Powered by Editorial Manager® and ProduXion Manager® from Aries Systems Corporation

	Meyrema Abdo Komicha, PhD candidate
**Response to Reviewers:**	First of all, thank you all, for your nice comments and suggestions, we try to correct the comments as the following:<br>1.We tried to edit our manuscript according to PLOS ONE's style requirements<br>2.Rephrased and cited overlapping text with the previous publication<br>3.Ethical statements are clarified in the document<br>4.The funding is more clarified as below:<br>Worku Dugassa Girsha wined the grant for the research. This study was entirely funded by the Oromia Regional Health Bureau. The total grant for the project was 156,000 Ethiopian birr (equivalent to 2,600 US$). The Oromia Regional Health Bureau funded only the indicated financial support, whereas, our institution, Adama hospital medical college provided vehicles with fuel for supervision. All authors are permanent workers of Adama hospital medical college and received regular monthly salaries, but not related to this study. Website of Oromia Regional Health Bureau: Email: ohbhead@ethionet.et. The funders had no role in study design, data collection, and analysis, decision to publish, or preparation of the manuscript.<br>5.The following Supporting information is provided<br>a.S1 File. (Information sheet, consent form, and English version questionnaires (PDF)<br>b.S1 Data (SPSS Data Set) (SAV)<br>6.For systematic sampling, sampling frame or list and Stratification statistics were clarified<br>7.For logistic regression, basic assumptions (normality, model fitness, and multicollinearity) were checked and revised (included in the manuscript)<br>8.Authors' contributions corrected according to guideline contents<br>9.A marked-up copy of our manuscript that highlighted changes with yellow color and an unmarked version of our revised paper without color changes are attached.<br>10. Laboratory protocols are not used or applied in the study and we are free of that.<br>With all the best regards<br>Worku Dugassa Girsha
**Additional Information:**	
**Question**	**Response**
**Financial Disclosure**<br><br>Enter a financial disclosure statement that describes the sources of funding for the work included in this submission. Review the submission guidelines for detailed requirements. View published research articles from *PLOS ONE* for specific examples.<br><br>This statement is required for submission and **will appear in the published article** if the submission is accepted. Please make sure it is accurate.	Worku Dugassa Girsha wined the grant for the research. This study was entirely funded by the Oromia Regional Health Bureau. The total grant for the project was 156,000 Ethiopian birr (equivalent to 2,600 US$), website of Oromia Regional Health Bureau: Email: The funders had no role in study design, data collection and analysis, decision to publish, or preparation of the manuscript.ohbhead@ethionet.et

**Unfunded studies**<br>Enter: *The author(s) received no specific funding for this work.*<br><br>**Funded studies**<br>Enter a statement with the following details:<br>• Initials of the authors who received each award<br>• Grant numbers awarded to each author<br>• The full name of each funder<br>• URL of each funder website<br>• Did the sponsors or funders play any role in the study design, data collection and analysis, decision to publish, or preparation of the manuscript?<br>• **NO** - Include this sentence at the end of your statement: *The funders had no role in study design, data collection and analysis, decision to publish, or preparation of the manuscript.*<br>• **YES** - Specify the role(s) played.<br><br>* typeset	
**Competing Interests**<br><br>Use the instructions below to enter a competing interest statement for this submission. On behalf of all authors, disclose any competing interests that could be perceived to bias this work—acknowledging all financial support and any other relevant financial or non-financial competing interests.<br><br>This statement is required for submission and **will appear in the published article** if the submission is accepted. Please make sure it is accurate and that any funding sources listed in your Funding Information later in the submission form are also declared in your Financial Disclosure statement.<br><br>View published research articles from *PLOS ONE* for specific examples.	The authors have declared that no competing interests exist.

**NO authors have competing interests**<br><br>Enter: *The authors have declared that no competing interests exist.*<br><br>**Authors with competing interests**<br><br>Enter competing interest details beginning with this statement:<br><br>*I have read the journal's policy and the authors of this manuscript have the following competing interests: [insert competing interests here]*<br><br>* typeset	
**Ethics Statement**<br><br>Enter an ethics statement for this submission. This statement is required if the study involved:<br><br>• Human participants<br>• Human specimens or tissue<br>• Vertebrate animals or cephalopods<br>• Vertebrate embryos or tissues<br>• Field research<br><br>Write "N/A" if the submission does not require an ethics statement.<br><br>General guidance is provided below. Consult the submission guidelines for detailed instructions. **Make sure that all information entered here is included in the Methods section of the manuscript.**	Ethical approval and clearance were obtained from the Adama Hospital Medical College, Institutional Ethical Review Committee. The health and administrative offices of the town received a letter of cooperation and ethical approval. Then, approval was obtained by contacting officials at various levels. The college institutional review board approved the information sheet and consent form, which was then attached to the questionnaire's first page. Before beginning to collect data, data collectors read each information sheet and consent form to enable mothers to make the right decisions. Furthermore, all the study participants were informed about the study's objective, benefits, and risks. Verbal consent of all study subjects was obtained before data collection. Participants were also informed that they have the full right to discontinue or refuse to participate in the study. To ensure confidentiality, the name of the interviewee wasn’t written on the questionnaire. Each respondent was assured that the information provided by them was confidential and used only for research. Moreover, no risk or harm was anticipated in the participation of the study.

Powered by Editorial Manager® and ProduXion Manager® from Aries Systems Corporation

**Format for specific study types**<br><br>**Human Subject Research (involving human participants and/or tissue)**<br>• Give the name of the institutional review board or ethics committee that approved the study<br>• Include the approval number and/or a statement indicating approval of this research<br>• Indicate the form of consent obtained (written/oral) or the reason that consent was not obtained (e.g. the data were analyzed anonymously)<br><br>**Animal Research (involving vertebrate animals, embryos or tissues)**<br>• Provide the name of the Institutional Animal Care and Use Committee (IACUC) or other relevant ethics board that reviewed the study protocol, and indicate whether they approved this research or granted a formal waiver of ethical approval<br>• Include an approval number if one was obtained<br>• If the study involved *non-human primates*, add *additional details* about animal welfare and steps taken to ameliorate suffering<br>• If anesthesia, euthanasia, or any kind of animal sacrifice is part of the study, include briefly which substances and/or methods were applied<br><br>**Field Research**<br><br>Include the following details if this study involves the collection of plant, animal, or other materials from a natural setting:<br>• Field permit number<br>• Name of the institution or relevant body that granted permission	
**Data Availability**<br><br>Authors are required to make all data underlying the findings described fully available, without restriction, and from the time of publication. PLOS allows rare exceptions to address legal and ethical concerns. See the PLOS Data Policy and FAQ for detailed information.	Yes - all data are fully available without restriction

A Data Availability Statement describing where the data can be found is required at submission. Your answers to this question constitute the Data Availability Statement and **will be published in the article**, if accepted.<br><br>**Important:** Stating 'data available on request from the author' is not sufficient. If your data are only available upon request, select 'No' for the first question and explain your exceptional situation in the text box.<br><br>Do the authors confirm that all data underlying the findings described in their manuscript are fully available without restriction?	
**Describe where the data may be found in full sentences. If you are copying our sample text, replace any instances of XXX with the appropriate details.**<br><br>• If the data are **held or will be held in a public repository**, include URLs, accession numbers or DOIs. If this information will only be available after acceptance, indicate this by ticking the box below. For example: *All XXX files are available from the XXX database (accession number(s) XXX, XXX.*).<br>• If the data are all contained **within the manuscript and/or Supporting Information files**, enter the following: *All relevant data are within the manuscript and its Supporting Information files.*<br>• If neither of these applies but you are able to provide **details of access elsewhere**, with or without limitations, please do so. For example:<br><br>*Data cannot be shared publicly because of [XXX]. Data are available from the XXX Institutional Data Access / Ethics Committee (contact via XXX) for researchers who meet the criteria for access to confidential data.*<br><br>*The data underlying the results presented in the study are available from (include the name of the third party*	All relevant data are within the manuscript and its Supporting Information files.

Powered by Editorial Manager® and ProduXion Manager® from Aries Systems Corporation

and contact information or URL).<br>• This text is appropriate if the data are owned by a third party and authors do not have permission to share the data.<br><br>* typeset	
Additional data availability information:	Tick here if the URLs/accession numbers/DOIs will be available only after acceptance of the manuscript for publication so that we can ensure their inclusion before publication.

Powered by Editorial Manager® and ProduXion Manager® from Aries Systems Corporation

Manuscript

Click here to access/download;Manuscript;Revised manuscript.docx

Indoor air pollution prevention practices and associated factors among household mothers in Olenchiti town, Oromia, Ethiopia

Worku Dugassa Girsha[1*], Alem Deksisa Abebe[1&], Ephrem Mannekulih Habtewold[1&], Meyrema Abdo Komicha[1&]

[1]Department of Public Health, Adama Hospital Medical College, Adama, Ethiopia

* Corresponding author

Email: dugassaworku@gmail.com (WD)

&- These authors also contributed equally to this work.

Abstract

Introduction: Most households in low- and middle-income countries still cook using solid fuels in poorly ventilated dwellings. Indoor air pollution causes various health problems, like pneumonia, lung cancer, stillbirth, low birth weight, impaired cognitive development, and cataracts. Nevertheless, a few evidences are available in Africa, including Ethiopia. Therefore, this study aimed to assess the level of indoor air pollution prevention practices and associated factors among household mothers in Olenchiti town, Oromia, Ethiopia.

Methods: A community-based cross-sectional study was conducted. Four hundred twenty mothers were randomly selected by systematic random sampling. Data was collected through an interview and observation checklist. The collected data entered into Epi-Info version 7.2.5 was cleaned, edited, and then exported to SPSS version 23 for analysis. Descriptive statistics were used to describe the findings. Binary logistic regression was computed to analyze the effect of each variable on the outcome variable. Model adequacy fitness was checked with the Hosmer-Lemeshow test. The multicollinearity of independent variables was checked with the variance inflation factor. Adjusted odds ratio with 95% confidence interval and P -value <0.05 was used as cutoff points to declare significance in the final model.

Results: The overall good practices of mothers towards indoor air pollution was 188 (45.0%). Mothers who had under-five children (AOR =0.49, 95%CI (0.31-0.76), mothers in grade 9 – 12 (AOR=0.51, 95%CI (0.28 - 0.92)), and diploma and above (AOR=0.28, 95%CI (0.09 - 0.84)) were significantly associated with indoor air pollution prevention practices.

Conclusion: The overall good practices of mothers towards indoor air pollution were low compared to different findings. Under-five children and educational status were significantly associated with indoor air pollution prevention practices in the final model. Therefore, the high school and college curriculums should include indoor air pollution topics.

Keywords: indoor air pollution, prevention practices, associated factors, Olanchity town, Ethiopia

Introduction

Most households in low- and middle-income countries still cook using solid fuels (such as wood, crop wastes, charcoal, coal, and dung) and kerosene in poorly ventilated dwellings [1,2]. Based on an analysis of household behavioral intervention practices of indoor air pollution (IAP), intervention should focus on the following behaviors: having separate kitchens, using modern stoves, keeping the room clean, use of clean energy sources, keeping children at least two meters away from burning fires, open windows for ventilation while fires are burning, reduce the duration of solid fuel burning and avoid indoor cigarette smoking [3,4].

Ensuring universal access to clean fuel and technologies is a target of the Sustainable Development Goal on Energy (SDG 7). However, Over half a billion people in Africa still rely on biomass fuel for cooking [5,6]. About 90% of the population of Ethiopia uses biomass fuels to meet household energy needs [7]. Indoor air pollution causes high risks for women and children due to their domestic roles [8,9]. Indoor air pollution causes various health problems, like pneumonia and acute lower respiratory infections [10,11], chronic obstructive pulmonary disease (COPD) [12], lung cancer [13], stillbirth or low birth weight [14], impaired cognitive development [15], cataracts [16] and nasopharyngeal and laryngeal cancers [12].

There is research evidence gaps in prevention practices of indoor air pollution. Most of the research finding in developing countries including Ethiopia focused on measuring the pollution level of pollutants and relating to diseases. However, only a few study evidence is available on indoor air pollution prevention practices. The level of indoor air pollution prevention practices conducted in different African countries showed that, in the rural village of South Africa, almost half the households had good practices of indoor air pollution prevention methods [17], Cameroon, (77%) of households reported exclusive use of firewood [18], in Kenya thirty-seven percent of observed houses had no windows [19,20], in Kasangati Town of Uganda, (11.5%) of respondents cooked inside their living houses [21].

In Ethiopia, different studies indicated that, in Northwest Ethiopia, (46%) [22] and rural Jimma zone, Ethiopia, (39%) [23] of households had good practices of indoor air pollution prevention methods. In Adama town, Ethiopia, (75.4%) of households used solid fuel for cooking in poorly ventilated houses [24]. Concerning factors affecting IAP, different studies conducted by the world health organization [25], in China [26], Bangladesh [27], rural Honduras [28], Kenya

[29], Uganda [30], and Ethiopia [31,32] revealed that income, education level, housing conditions, main fuel source and long hours burning solid fuels were significantly associated with the level of indoor air pollution practices.

Like other rural towns in Ethiopia, poorly ventilated housing, and limited access to separate cooking are visible features of living conditions in Olanchity town. Nevertheless, the present level of indoor air pollution prevention practices has not been studied in Olanchity town. Thus, the results of the study are used to create awareness for households, especially mothers to improve the efficient utilization of their household energy. It also helps the concerned governmental and nongovernmental organizations to design appropriate intervention strategies. Besides, it will be used by other interested researchers as a reference. Therefore, the study aims to assess the level of indoor air pollution prevention practices and associated factors among household mothers in the study area.

Materials and methods

Study area

The study was carried out in Olenchiti town, East Shoa zone, Orom5ia region, Ethiopia. The town is located 25 km from Adama town, along the road from Addis Ababa to Dire Dawa [33]. The town has two kebeles with a total population of 32,638 and 8,028 households. The town has one governmental hospital and health center.

Study design and period

A community-based cross-sectional study design was conducted from the 1st of November 2022 to the 30th of April 2023.

Source and study population

The source population includes all household mothers in Welenchiti Town. The study population was all household mothers in the selected kebele of Welenchiti town. Households with only a mother of age greater than or equal to 18 years were included in the study.

Sample size determination and sampling procedures

The following assumptions were considered to determine the sample size: from a study done in Northwest Ethiopia, mothers had 46% indoor air pollution prevention practices [22]. Level of confidence, 95%, (Zα/2) = 1.96, A 5% margin of error (d=0.05). Additional 10 % for non-response rate. Then sample size was calculated by using the formula estimate of single population proportion:

$$n = \frac{(Z_{\alpha/2})^2 * P(1-P)}{d^2} = \frac{(1.96)^2 * (0.46) * (0.54)}{(0.05)^2} = 382$$

Then adding an estimated 10% for the non-respondent rate the final sample size was 420. The town was stratified into kebeles to select a fairly representative sample of mothers from households. The sample size was distributed proportionally to each of the kebeles based on the number of households they have. Kebele one had a total of 5803 households (proportional allocation 304) and Kebele two had a total of 2225 households (proportional allocation of 116). After assigning a number to each house and obtaining a sampling frame, each household sample was selected by systematic random sampling. The next household was substituted for households that didn't have the required mother. However, for households with more than one mother, the mother of the family representative was given priority, otherwise, selected by the lottery method.

Study variables and data collection techniques

The dependent variable was indoor air pollution prevention practices and the independent variables include, socio-demographic factors (age, family size, under-five children, educational status, monthly income, marital status, occupation), housing-related factors (separate kitchen, presence of window, presence of chimney, type of stove, type of roof construction), household's behavioral factors (cigarette smoking, duration of cooking, complete combustion, opening window, knowledge on effects of indoor air pollution, training, supervision) and household's sources of energy (electricity, wood, charcoal, dung, kerosene).

An interviewer-administered semi-structured questionnaire adapted and modified from different literature was used to collect data with an observation checklist. The data were collected by face-to-face interviews through house-to-house observation.

Data collection was administered by four BSc in environmental health professionals and two supervisors who have a general master of public health.

Data quality assurance and measurements

A five percent pre-test of the instrument was done in Adama town. During the pre-test, a discussion was held with the interviewers on the problems they encountered to collect data. The correction was incorporated in the final questionnaire. The training was given to both data collectors and supervisors a day before the pretest and a day after the pretest. The training includes the objectives of the study, method of data collection, checking completeness of the questionnaire, and the way to approach the households. Adjusted analysis was performed to account for potential confounders. Finally, Continuous supervision was done.

Indoor air pollution prevention practices were categorized into good practice or poor practice by considering the percentage of mothers who correctly answered or fulfilled the given six questionnaires such as the use of a separate kitchen, opening a window while cooking, use of a modern stove, outside complete combustion of charcoal, separating under five children while cooking, and use of clean energy sources.

Data processing and analysis

The collected data were checked, entered into Epi-Info version 7.2.5, cleaned, edited, and then exported to SPSS version 23.0 for analysis. Descriptive statistics were used to describe the findings. The normality of the continuous variable was checked with Shapiro-Wilk Test (P<0.01). Binary logistic regression was computed to analyze the effect of each variable on the outcome variable. During bivariate logistic regression, independent variables with a P _value of <0.25 were considered candidates for the final logistic regression model. Then all candidate variables were subjected to multiple regression analysis to identify factors significantly associated with indoor air pollution practices after adjusting for possible confounding variables in the finally fitted regression model. Hosmer and Lemeshow test was performed to evaluate the model goodness of fit by comparing the disparities between observed and predicted values. The diagnosis results revealed that the model fit the data adequately and there was no significant difference between observed and expected values (P-value = 0.44). The variance inflation factors (VIF) were used to assess if

there were multicollinearity among covariates in the fitted model. The results of diagnosis showed that none of the covariates showed the VIF value above five which shows the absence of collinearity among covariates. The strength of association was expressed in an adjusted odds ratio with 95% confidence interval and P -value <0.05 was used as cutoff points to declare the statistical significance in the final model.

Ethical considerations

Ethical approval and clearance were obtained from the Adama Hospital Medical College, Institutional Ethical Review Committee. The health and administrative offices of the town received a letter of cooperation and ethical approval. Then, approval was obtained by contacting officials at various levels. The college institutional review board approved the information sheet and consent form, which was then attached to the questionnaire's first page. Before beginning to collect data, data collectors read each information sheet and consent form to enable mothers to make the right decisions. Furthermore, all the study participants were informed about the study's objective, benefits, and risks. Verbal consent of all study subjects was obtained before data collection. Participants were also informed that they have the full right to discontinue or refuse to participate in the study. To ensure confidentiality, the name of the interviewee wasn’t written on the questionnaire. Each respondent was assured that the information provided by them was confidential and used only for research. Moreover, no risk or harm was anticipated in the participation of the study.

Results

Socio-demographic characteristics of respondents

A total of 422 mothers were interviewed with a response rate of 99.52%. The median age was 37 years with an interquartile range of 16 years. The minimum and maximum age was 18 and 82 years respectively. One hundred forty-three (34.2%) of them founded in the age category of 31 – 40 years. One hundred eighty-five (44.3%) households had under five years children. Among the respondents, 284 (67.9%) were Oromo by ethnicity and 180 (43.1%) were Orthodox in religion. Regarding educational status, 105 (25.1%) had elementary (grade 1-8) levels and 192 (45.9%) were housewives in occupation. Regarding household monthly income, 179 (42.8%) got less than 36 US$ per month (Table 1).

Table 1: Socio-demographic Characteristics among households in Olanchitiy town, East Shewa zone, Oromia, Ethiopia, 2023

**Variables (n=418)**	**Number (%)**
Family size	
< 5	284 (67.9)
≥ 5	134 (32.1)
Having under-five children	
Yes	185 (44.3)
No	233 (55.7)
Age of respondents	
18 – 30	133 (31.8)
31 -40	143 (34.2)
41 -50	89 (21.3)
51 and above	53 (12.7)
Ethnicity	
Oromo	284 (67.9)
Amhara	118 (28.2)
Gurage	14 (3.3)
Others*	2 (0.5)
Religion	
Muslim	153 (36.6)
Orthodox	180 (43.1)
Protestant	80 (19.1)
Waqefata	5 (1.2)
Educational status	
Unable to read and write	104 (24.9)
Read and write	71 (17.0)
Grade 1-8	105 (25.1)
Grade 9 -12	91 (21.8)
Diploma and above	47 (11.2)
**Occupation**	
Housewife	192 (45.9)
Merchant	94 (22.5)
Governmental employee	33 (7.9)
Daily laborer	31 (7.4)

Private employee	39 (9.3)
Farmer	29 (6.9)
Household monthly income (ETB)	
<2000	179 (42.8)
2001 – 4000	161 (38.5)
4001 – 5000	37 (8.9)
5001 and above	41 (9.8)

*=Tigire and Kembata

Housing conditions-related factors of households

Two hundred ninety-nine (71.5%) households had a separate kitchen from the main living room and 110 (26.3%) of household's kitchens or living rooms had windows and 122 (29.2%) of them had a chimney vent pipe for ventilation. One hundred fifty-four (36.8%) households used modern stoves for cooking and 291 (69.6%) of their roof were constructed with corrugated iron sheets (Table 2).

Table 2: Housing conditions-related factors among households in Olanchitiy town, East Shewa zone, Oromia, Ethiopia, 2023

**Variables (n=418)**	**Number (%)**
Having a separate kitchen from the living room	
Yes	299 (71.5)
No	119 (28.5)
The kitchen or living room had a window	
Yes	110 (26.3)
No	308 (73.3)
The housing had a Chimney (vent pipe)	
Yes	122 (29.2)
No	296 (70.8)
Having a modern stove for cooking	
Yes	154 (36.8)
No	264 (63.2)
Type of modern stoves (n=154)	
Mixadi*	50 (32.5)
Electric stove	73 (47.4)

Midija (lakech)*	31 (20.1)
Type of roof construction	
Corrugated Iron sheet	291 (69.6)
Traditional thatch roof	125 (29.6)
Others (plastic)	2 (0.5)
Room cleanliness (dust)	
Cleaned	248 (59.3)
Not cleaned	170 (40.7)

*=Ethiopian locally-made modern stoves

Household's Sources of energy

Among the studied households, Only 131 (31.3%) were using electricity for cooking. However, most (99.3%) households used wood or charcoal for food preparation. Similarly, almost all (99%) households were using mixed sources of energy such as electricity, wood, charcoal, animal dung, and kerosene (Table 3)

Table 3: Household's sources of energy for cooking in Olanchitiy town, East Shewa zone, Oromia, Ethiopia, 2023

Variables (n=418)	Number (%)
Electricity	
Yes	131 (31.3)
No	287 (68.7)
Wood	
Yes	415 (99.3)
No	3 (0.7)
Charcoal	
Yes	415 (99.3)
No	3 (0.7)
Dung	
Yes	242 (57.9)
No	176 (42.1)
Kerosene	
Yes	35 (8.4)
No	383 (91.6)

Mixed sources utilization	
Yes	414 (99.0)
No	4 (1.0)

Household’s behavioral factors

Only 19 (4.5%) of household members practiced indoor cigarette smoking. Two hundred ninety-two (69.9%) households opened their window or door while cooking. Around nine-tenths, (90.2%) of families did outside complete combustion of charcoal before entering the house. Only 69 (16.5%) households separated under five children while cooking (at least 2m from the burning fire). Most mothers (90.9%) continuously cooked in the kitchen for more than one hour without going outside the kitchen to take fresh air. Only a few mothers (2.6%) got training or follow-up on IAP from the health or energy bureau during the last six months. Forty-four households (10.5%) reported diseases during the last two weeks. Among the reported diseases, respiratory diseases accounted for 27 (61.4%) (Table 4).

Table 4: Household’s behavioral factors on IAP in Olanchitiy town, East Shewa zone, Oromia, Ethiopia, 2023

**Variables (n=418)**	**Number (%)**
Indoor Cigarette smoking among households	
Yes	19 (4.5)
No	399 (95.5)
Opening window or door while cooking	
Yes	292 (69.9)
No	126 (30.1)
Outside Complete combustion of charcoal before entering the house	
Yes	377 (90.2)
No	41 (9.8)
Separation of under-five children while cooking	
Yes	69 (16.5)
No	349 (83.5)
Cooking in the kitchen for more than 1hrs without going out	
Yes	380 (90.9)
No	38 (9.1)

Training or follow-up was given on IAP by the health or energy bureau (6 months)	
Yes	11 (2.6)
No	407 (97.4)
Diseases reported among HH during the last two weeks	
Yes	44 (10.5)
No	418 (89.5)
Type of diseases reported	
Respiratory diseases	27 (61.4)
Eye diseases	13 (29.5)
Cancer	4 (9.1)

Knowledge of Indoor Air Pollution

Most of the mothers, 386 (92.3%) and 406 (97.1%) mentioned indoor air pollution as a cause of respiratory diseases and cataracts or eye problems respectively. Some mothers also mentioned indoor air pollution as a cause of some health and related problems: lung cancer 262 (62.7%), low birth weight 220 (52.6%), impaired cognitive development 214 (51.2%), and global climate change 288 (68.9%). Concerning the overall knowledge of mothers, 279 (66.7%) had good knowledge of the impact of indoor air pollution (Table 5).

Table 5: Knowledge of IAP among mothers in Olanchitiy town, East Shewa zone, Oromia, Ethiopia, 2023

**Variables (n=418)**	**Number (%)**
IAP causes respiratory diseases	
Yes	386 (92.3)
No	32 (7.7)
IAP causes lung cancer	
Yes	262 (62.7)
No	156 (37.3)
IAP causes low birth weight	
Yes	220 (52.6)
No	198 (47.4)
IAP Impaired cognitive development	
Yes	214 (51.2)

No	204 (48.8)
IAP causes cataract	
Yes	406 (97.1)
No	12 (2.9)
IAP causes global climate change	
Yes	288 (68.9)
No	130 (31.1)
Overall knowledge	
Good Knowledge	279 (66.7)
Poor knowledge	139 (33.3)

Household's indoor air pollution prevention practices

The overall good practices of households towards indoor air pollution were 188 (45.0%) with 95%CI (40.2 – 51.0) (Table 6)

Table 6: Household's IAP prevention practices in Olanchitiy town, East Shewa zone, Oromia, Ethiopia, 2023

**Variables (n=418)**	**Number (%)**
Use of separate kitchen	
Yes	299 (71.5)
No	119 (28.5)
Use of modern stove	
Yes	154 (36.8)
No	264 (63.2)
Opening window or door while cooking	
Yes	292 (69.9)
No	126 (30.1)
Separating under five children while cooking (at least 2m away from burning fires)	
Yes	69 (16.5)
No	349 (83.5)
Use of clean energy source (electricity)	
Yes	131 (31.3)
No	287 (68.7)

Outside complete combustion of charcoal	
Yes	377 (90.2)
No	41 (9.8)
Overall practice	
Good Practices	188 (45.0)
Poor Practices	230 (55.0)

Factors affecting household's indoor air pollution prevention practices

In the binary logistic regression variables such as family size, under-five children, age of respondents, educational status, occupation, household monthly income, and training were statistically significant at p-value < 0.25 and transferred to the multivariable analysis. However, after adjusting for possible confounding factors, only under-five children's and educational status were associated with indoor air pollution prevention practices at p-value ≤ 0.05.

Among households who had under-five children 48 % (AOR =0.52, 95%CI (0.33-0.82) were less likely to practice indoor air pollution prevention practices than those who didn't have under-five children. Similarly, mothers in grade 9 – 12 educational level 49% (AOR=0.51, 95%CI (0.28 - 0.92)) and diploma and above were 72% (AOR=0.28, 95%CI (0.09 - 0.84)) less likely to practice indoor air pollution prevention practices than those unable to read and write (Table 7).

Table 7: Factors affecting household IAP prevention practices in Olanchitiy town, East Shewa zone, Oromia, Ethiopia, 2023

**Variables**	**IAP prevention practices**		**COR (95%CI)**	**AOR (95%CI)**	**P - value**
	**Good N (%)**	**Poor N (%)**			
Family size					
< 5	111(39.1)	173(60.9)	2.10 (1.38 -3.19)	1.37(0.83-2.26)	0.20
≥ 5	77(57.5)	57(42.5)	1.0	1.0	
Under five children					
Yes	106(57.3)	79(42.7)	0.40(0.27 – 0.60)	**0.52(0.33-0.82)**	0.01
No	82(35.2)	151(64.8)	1.0	1.0	

Age of respondents					
18 – 30	49(36.8)	84(63.2)	0.95 (0.49 -1.85)	1.18(0.54-2.59)	0.67
31 -40	73(51.0)	70(49.0)	0.53 (0.28 – 1.02)	0.64(0.30-1.37)	0.25
41 -50	47(52.8)	42(47.2)	0.49 (0.24 – 1.00)	0.56(0.26-1.22)	0.15
51 and above	19(35.8)	34(64.2)	1.0	1.0	
Educational status					
Illiterate	36(34.6)	68(65.4)	1.0	1.0	
Read and write	27(38.0)	44(62.0)	0.86 (0.46 -1.61)	0.71 (0.36- 1.40)	0.33
Grade 1-8	43(41.0)	62(59.0)	0.76 (0.43-1.33)	0.73 (0.40 - 1.34)	0.32
Grade 9 -12	48(52.7)	43(47.3)	0.47 (0.26 -0.84)	**0.45 (0.24 - 0.88)**	**0.01**
Diploma and above	34(72.3)	13(27.7)	0.20 (0.09 -0.43)	**0.28 (0.09 -0.84)**	**0.02**
Occupation					
Housewife	82(42.7)	110(57.3)	0.51 (0.21 - 1.21)	0.56(0.23-1.37)	0.20
Merchant	50(53.2)	44(46.8)	0.33 (0.13 -0.83)	0.49(0.18-1.28)	0.14
Governmental employee	26(78.8)	7(21.2)	0.10 (0.03 – 1.32)	0.42(0.09-1.78)	0.24
Daily laborer	7(22.6)	24(77.4)	1.30 (0.40 - 4.21)	1.37(0.40-4.70)	0.61
Private employee	15(38.5)	24(61.5)	0.61 (0.21 – 1.72)	1.01(0.33-3.08)	0.98
Farmer	8(27.6)	21(72.4)	1.0	1.0	
Household monthly income (ETB)					
<2000	67(37.4)	112(62.6)	1.0	1.0	
2001 – 4000	74(46.0)	87(54.0)	0.70 (0.45- 1.08)	0.82 (0.51-1.33)	0.43
4001 - 5000	17(45.9)	20(54.1)	0.70 (0.34 -1.43)	1.14 (0.51-2.57)	0.73
5001 and above	30(73.2)	11(26.8)	0.21 (0.10 -0.46)	0.48 (0.19 – 1.17)	0.11
Training					
Yes	7(63.6)	4(36.4)	0.45 (0.13 – 1.58)	0.48(0.11-1.97)	0.31
No	181(44.5)	226(55.5)	1.0	1.0	

Abbreviations: COR crude odds ratio, AOR adjusted odds ratio, CI confidence interval, 1.0 reference category

Discussions

The study assessed the level of indoor air pollution prevention practices and associated factors among households in Olenchiti town, East Shewa zone, Oromia, Ethiopia. Based on the main findings, less than half of the households

practiced good indoor air pollution prevention, (45.0%, 95%CI (40.2 – 51.0)). The finding was similar to a study done in a rural village in South Africa, 51% [17] and in Northwest Ethiopia, (46%) [22], however, higher than the study done in rural Jimma, (39%) [23], the difference may be due to the study time variations and methods.

Regarding household energy sources, most (68.7%) households used biomass fuel for cooking. The finding was almost similar to a World Bank report, in Africa, over half a billion people still rely on biomass fuel for cooking and heating [6]. This finding was much behind the target of the Sustainable Development Goal on Energy (SDG 7).which says to ensure universal access to clean fuel and technologies.

Concerning the housing condition of households, (28.5%) households had no separate kitchen from the main living room and cooked inside their living houses. The finding was similar to a study done in a rural town in Kenya (37%) [20], however, the finding was higher than the study done in a study conducted in rural Town of Uganda (11.5%) [21], with a slight difference may be due to study time variation and methods. Similarly. Only (36.8%) of households used improved or modern stoves for cooking, almost similar to a study done in a rural village in South Africa (27%) [17]. Regarding household behavioral factors, (69.9%) of households opened windows or doors while cooking. Similar to a study done in a rural village in South Africa (69%) [17], the possible reasons may be the knowledge of mothers regarding the impact of indoor air pollution on the household members should be increased.

Households that had under-five children were 51 % less likely to practice indoor air pollution prevention practices than those who didn’t have under-five children. Many findings suggested that mothers with under five children are so busy, much of their time is wasted on child caring, cooking, family guidance, and income generation. Similarly, mothers in grades 9 – 12 educational level (49%) and diploma and above (72%) were less likely to practice indoor air pollution prevention practices than those unable to read and write. The finding was contrary to studies done in China [26] and Bangladesh [27] which suggested that households with highly educated adults practice IAP prevention more likely than the least-educated households. The difference may be in the study area, about IAP topics not included in their high school and college curriculum and attention was not given to high school and college students.

Limitations of the Study

The study findings included household mothers from a single town. Therefore, the research results are limited to this particular town and cannot be generalized to other towns in Ethiopia.

Conclusions and recommendations

The overall good practices of households towards indoor air pollution were low compared to different findings in Ethiopia as well as in Africa. Under-five children and educational status were significantly associated with indoor air pollution prevention practices in the final model. Therefore, this study recommends the following actions to alleviate indoor air pollution problems in Welenchiti town. The town health office should be strengthened family planning services for households to regulate their family size. Similarly, the town educational offices should be incorporated indoor air pollution topics into regular high school and college curriculums. Further research is needed for measuring the concentration level of indoor air pollutants.

Acknowledgments

We hugely acknowledge the different levels of administrative hierarchy. Furthermore, the authors like to acknowledge the study participants, data collectors, and colleagues for their genuine cooperation during the course of the study.

References

1 Fullerton DG, Semple S, Kalambo F, Suseno A, Malamba R, Henderson G,et al. Biomass fuel use and indoor air pollution in homes in Malawi. Occup Environ Med, 2009

2 Langbein J. Firewood, smoke and respiratory diseases in developing countries: the neglected role of outdoor cooking, 2017, https://www.ncbi.nlm.nih.

3 Ferguson, et al. Exposure to indoor air pollution across socio-economic groups in high income countries: A scoping review of the literature and a modelling methodology, Environment International, 2020, https://doi.org/10.1016/j.envint.2020.105748

4 Barnes et al, A behavioral intervention to reduce child exposure to indoor air pollution: identifying possible target behaviors. Health Education and Behavior, in press, 2004

5 IEA. World energy outlook. Paris: International Energy Agency (IEA); 2012. https://www.iea.org/topics/world-energy-outlook,

6 Ekouevi K, Tuntivate V. Household energy access for cooking and heating: lessons learned and the way forward: World Bank publications; 2012. https://doi.org/10.1596/978-0-8213-9604-9

7 Worku Tefera et al, Indoor and Outdoor Air Pollution- related Health Problem in Ethiopia: Review of Related Literature, Article in Ethiopian Journal of Health Development, 2016 https://www.researchgate.net/publication/313083044

8 Lim SS, Vos T, Flaxman AD, Danaei G, Shibuya K, et al. A comparative risk assessment of burden of disease and injury attributable to risk factors and risk factor clusters in 21 regions: a systematic analysis for the Global Burden of Disease Study, 2012.

9 Women's Commission for Refugee Women and Children Beyond firewood: Fuel alternatives and protection strategies for displaced women and girls, New York, 2006.

10 UNEP Near-term climate protection and clean air benefits: actions for controlling short lived climate forcers. A UNEP synthesis report. Nairobi: UNEP 2011.

11 Shen M, Chapman RS, Vermeulen R, Tian L, Zheng T, et al. Coal use, stove improvement, and adult pneumonia mortality in Xuanwei, China: a retrospective cohort study, 2009.

12 Chapman RS, He X, Blair AE, Lam Q, Improvement in household stoves and risk of chronic obstructive pulmonary disease in Xuanwei, China: retrospective cohort study. BMJ, 2005.

13 Lan Q, Chapman RS, Schreinemachers DM, Tian L, He X, Household stove improvement and risk of lung cancer in Xuanwei, China, 2002.

14 Pope DP, Mishra VK, Thompson L, Siddiqui AR, Rehfuess E, et al. Risk of low birth weight and stillbirth associated with indoor air ollution from solid fuel use in developing countries, 2010

15 Dix-Cooper L, Eskenazi B, Romero C, Balmes J, Smith KR, Neurodevelopmental performance among school age children in rural Guatemala is associated with prenatal and postnatal exposure to carbon monoxide, a marker for exposure to woodsmoke, 2012

16 Pokhrel AK, Smith KR, Khalakdina A, Deuja A, Bates MN, Case-control study of indoor cooking smoke exposure and cataract in Nepal and India, 2005

17 B. R. Barnes et al. Testing selected behaviors to reduce indoor air pollution exposure in young children in a rural village of South Africa, Health Education Research Vol.19 no.5, Oxford University Press 2004

18 Esong et al. Distribution of sources of household air pollution: a cross-sectional study in Cameroon, BMC Public Health (2021) 21:318 https://doi.org/10.1186/s12889-021-103506

19 Dida et al. Factors predisposing women and children to indoor air pollution in rural villages, Western Kenya, Archives of Public Health BMC, (2022) 80:46. https://doi.org/10.1186/s13690-022-00791-9

20 Wilkister Nyaora Moturi, Risk factors for indoor air pollution in rural households in Mauche division, Molo district, Kenya, African Health Sciences Vol 10 No 3 September 2010

21 Serunjogi, D.T. and Musoke, D. Risk Factors for Indoor Air Pollution Exposure in Households in Kasangati Town Council, Wakiso District, Uganda, African Journal of Environmental Health Sciences, Volume 7, November, 2020 http://www.ehsanonline.org

22 Adane et al. Biomass-fuelled improved cook stove intervention to prevent household air pollution in Northwest Ethiopia: a cluster randomized controlled trial, Environmental Health and Preventive Medicine (2021), https://doi.org/10.1186/s12199-020-00923

23 Faris K. Survey of Indoor Air Pollution Problems in the Rural Communities of Jimma, Southwest Ethiopia. Ethiop J Health Sci. 2002; 12(1):1–8.

24 Balidemaj, F.; Isaxon, C.; Abera, A.; Malmqvist, E. Indoor Air Pollution Exposure of Women in Adama, Ethiopia, and Assessment of Disease Burden Attributable to Risk Factor. Int. J. Environ. Res. Public Health 2021, 18, 9859. https://doi.org/10.3390/ ijerph18189859

25 K. R. Smith and S. Mehta, "The burden of disease from indoor air pollution in developing countries: comparison of estimates," International Journal of Hygiene and Environmental Health, vol. 206, no. 4-5, pp. 279–289, 2003

26 Maya Papineau et al, Distributional determinants of household air pollution in China, Cambridge University Press, 2009. https://www.cambridge.org/core,

27 Susmita et al, who suffers from indoor air pollution? Evidence from Bangladesh, Published by Oxford University Press in association with The London School of Hygiene and Tropical Medicine, 2006.

28 Audrey Le et al. Perception and Barriers to Indoor Air Quality and Perceived Impact on Respiratory Health: An Assessment in Rural Honduras, Hindawi Publishing Corporation Advances in Public Health Volume 2014, http://dx.doi.org/10.1155/2014/105260

29 Dida et al. Factors predisposing women and children to indoor air pollution in rural villages, Western Kenya, Archives of Public Health BMC, 2022, 80:46. https://doi.org/10.1186/s13690-022-00791-9

30 E. Coker, et al. Household air pollution profiles associated with persistent childhood cough in urban Uganda, Environment International, 2020, https://doi.org/10.1016/j.envint.2020.105471

31 Dyjack D, Soret S, Chen L, Hwang R, Nazari N, Gaede D. Re sidential environmental risks for reproductive age women in developing countries. J Midwifery Womens Health, 2005

32 Central Statistical Authority (CSA) Ethiopia. Statistical Abstract, Addis Ababa, Ethiopia, 2015

33 Girsha W , Adlo A, Garoma D, Beggi S. Assessment of Water, Sanitation and Hygiene Status of Households in Welenchiti Town, Boset Woreda, East Shoa Zone, Ethiopia. Science Journal of Public Health, 2016. doi: 10.11648/j.sjph.20160406.13

Supporting information

S1 File. (PDF)

S1 Data Set. (SAV)

Click here to access/download
Supporting Information
Ethical Letter.pdf

Click here to access/download
Supporting Information
S1 Data.sav

Click here to access/download
Supporting Information
S1 File..pdf

Revised Manuscript with Track Changes

Indoor air pollution prevention practices and associated factors among household mothers in Olenchiti town, Oromia, Ethiopia

Worku Dugassa Girsha[1*], Alem Deksisa Abebe[1&], Ephrem Mannekulih Habtewold[l&], Meyrema Abdo Komicha[1&]

[1]Department of Public Health, Adama Hospital Medical College, Adama, Ethiopia

* Corresponding author

Email: dugassaworku@gmail.com (WD)

&- These authors also contributed equally to this work.

Abstract

Introduction: Most households in low- and middle-income countries still cook using solid fuels in poorly ventilated dwellings. Indoor air pollution causes various health problems, like pneumonia, lung cancer, stillbirth, low birth weight, impaired cognitive development, and cataracts. Nevertheless, a few evidences are available in Africa, including Ethiopia. Therefore, this study aimed to assess the level of indoor air pollution prevention practices and associated factors among household mothers in Olenchiti town, Oromia, Ethiopia.

Methods: A community-based cross-sectional study was conducted. Four hundred twenty mothers were randomly selected by systematic random sampling. Data was collected through an interview and observation checklist. The collected data entered into Epi-Info version 7.2.5 was cleaned, edited, and then exported to SPSS version 23 for analysis. Descriptive statistics were used to describe the findings. Binary logistic regression was computed to analyze the effect of each variable on the outcome variable. Model adequacy fitness was checked with the Hosmer-Lemeshow test. The multicollinearity of independent variables was checked with the variance inflation factor. Adjusted odds ratio with 95% confidence interval and P -value <0.05 was used as cutoff points to declare significance in the final model.

Results: The overall good practices of mothers towards indoor air pollution was 188 (45.0%). Mothers who had under-five children (AOR =0.49, 95%CI (0.31-0.76), mothers in grade 9 – 12 (AOR=0.51, 95%CI (0.28 - 0.92)), and diploma and above (AOR=0.28, 95%CI (0.09 - 0.84)) were significantly associated with indoor air pollution prevention practices.

Conclusion: The overall good practices of mothers towards indoor air pollution were low compared to different findings. Under-five children and educational status were significantly associated with indoor air pollution prevention practices in the final model. Therefore, the high school and college curriculums should include indoor air pollution topics.

Keywords: indoor air pollution, prevention practices, associated factors, Olanchity town, Ethiopia

Introduction

Most households in low- and middle-income countries still cook using solid fuels (such as wood, crop wastes, charcoal, coal, and dung) and kerosene in poorly ventilated dwellings [1,2]. Based on an analysis of household behavioral intervention practices of indoor air pollution (IAP), intervention should focus on the following behaviors: having separate kitchens, using modern stoves, keeping the room clean, use of clean energy sources, keeping children at least two meters away from burning fires, open windows for ventilation while fires are burning, reduce the duration of solid fuel burning and avoid indoor cigarette smoking [3,4].

Ensuring universal access to clean fuel and technologies is a target of the Sustainable Development Goal on Energy (SDG 7). However, Over half a billion people in Africa still rely on biomass fuel for cooking [5,6]. About 90% of the population of Ethiopia uses biomass fuels to meet household energy needs [7]. Indoor air pollution causes high risks for women and children due to their domestic roles [8,9]. Indoor air pollution causes various health problems, like pneumonia and acute lower respiratory infections [10,11], chronic obstructive pulmonary disease (COPD) [12], lung cancer [13], stillbirth or low birth weight [14], impaired cognitive development [15], cataracts [16] and nasopharyngeal and laryngeal cancers [12].

There is research evidence gaps in prevention practices of indoor air pollution. Most of the research finding in developing countries including Ethiopia focused on measuring the pollution level of pollutants and relating to diseases. However, only a few study evidence is available on indoor air pollution prevention practices. The level of indoor air pollution prevention practices conducted in different African countries showed that, in the rural village of South Africa, almost half the households had good practices of indoor air pollution prevention methods [17], Cameroon, (77%) of households reported exclusive use of firewood [18], in Kenya thirty-seven percent of observed houses had no windows [19,20], in Kasangati Town of Uganda, (11.5%) of respondents cooked inside their living houses [21].

In Ethiopia, different studies indicated that, in Northwest Ethiopia, (46%) [22] and rural Jimma zone, Ethiopia, (39%) [23] of households had good practices of indoor air pollution prevention methods. In Adama town, Ethiopia, (75.4%) of households used solid fuel for cooking in poorly ventilated houses [24]. Concerning factors affecting IAP, different studies conducted by the world health organization [25], in China [26], Bangladesh [27], rural Honduras [28], Kenya

[29], Uganda [30], and Ethiopia [31,32] revealed that income, education level, housing conditions, main fuel source and long hours burning solid fuels were significantly associated with the level of indoor air pollution practices.

Like other rural towns in Ethiopia, poorly ventilated housing, and limited access to separate cooking are visible features of living conditions in Olanchity town. Nevertheless, the present level of indoor air pollution prevention practices has not been studied in Olanchity town. Thus, the results of the study are used to create awareness for households, especially mothers to improve the efficient utilization of their household energy. It also helps the concerned governmental and nongovernmental organizations to design appropriate intervention strategies. Besides, it will be used by other interested researchers as a reference. Therefore, the study aims to assess the level of indoor air pollution prevention practices and associated factors among household mothers in the study area.

Materials and methods

Study area

The study was carried out in Olenchiti town, East Shoa zone, Orom5ia region, Ethiopia. The town is located 25 km from Adama town, along the road from Addis Ababa to Dire Dawa [33]. The town has two kebeles with a total population of 32,638 and 8,028 households. The town has one governmental hospital and health center

Study design and period

A community-based cross-sectional study design was conducted from the 1st of November 2022 to the 30th of April 2023.

Source and study population

The source population includes all household mothers in Welenchiti Town. The study population was all household mothers in the selected kebele of Welenchiti town. Households with only a mother of age greater than or equal to 18 years were included in the study.

Sample size determination and sampling procedures

The following assumptions were considered to determine the sample size: from a study done in Northwest Ethiopia, mothers had 46% indoor air pollution prevention practices [22]. Level of confidence, 95%, (Zα/2) = 1.96, A 5% margin of error (d=0.05). Additional 10 % for non-response rate. Then sample size was calculated by using the formula estimate of single population proportion:

$$n = \frac{(Z_{\alpha/2})^2 * P(1-P)}{d^2} = \frac{(1.96)^2 * (0.46) * (0.54)}{(0.05)^2} = 382$$

Then adding an estimated 10% for the non-respondent rate the final sample size was 420. The town was stratified into kebeles to select a fairly representative sample of mothers from households. The sample size was distributed proportionally to each of the kebeles based on the number of households they have. Kebele one had a total of 5803 households (proportional allocation 304) and Kebele two had a total of 2225 households (proportional allocation of 116). After assigning a number to each house and obtaining a sampling frame, each household sample was selected by systematic random sampling. The next household was substituted for households that didn't have the required mother. However, for households with more than one mother, the mother of the family representative was given priority, otherwise, selected by the lottery method.

Study variables and data collection techniques

The dependent variable was indoor air pollution prevention practices and the independent variables include, socio-demographic factors (age, family size, under-five children, educational status, monthly income, marital status, occupation), housing-related factors (separate kitchen, presence of window, presence of chimney, type of stove, type of roof construction), household's behavioral factors (cigarette smoking, duration of cooking, complete combustion, opening window, knowledge on effects of indoor air pollution, training, supervision) and household's sources of energy (electricity, wood, charcoal, dung, kerosene).

An interviewer-administered semi-structured questionnaire adapted and modified from different literature was used to collect data with an observation checklist. The data were collected by face-to-face interviews through house-to-house observation.

Data collection was administered by four BSc in environmental health professionals and two supervisors who have a general master of public health.

Data quality assurance and measurements

A five percent pre-test of the instrument was done in Adama town. During the pre-test, a discussion was held with the interviewers on the problems they encountered to collect data. The correction was incorporated in the final questionnaire. The training was given to both data collectors and supervisors a day before the pretest and a day after the pretest. The training includes the objectives of the study, method of data collection, checking completeness of the questionnaire, and the way to approach the households. Adjusted analysis was performed to account for potential confounders. Finally, Continuous supervision was done.

Indoor air pollution prevention practices were categorized into good practice or poor practice by considering the percentage of mothers who correctly answered or fulfilled the given six questionnaires such as the use of a separate kitchen, opening a window while cooking, use of a modern stove, outside complete combustion of charcoal, separating under five children while cooking, and use of clean energy sources.

Data processing and analysis

The collected data were checked, entered into Epi-Info version 7.2.5, cleaned, edited, and then exported to SPSS version 23.0 for analysis. Descriptive statistics were used to describe the findings. The normality of the continuous variable was checked with Shapiro-Wilk Test (P<0.01). Binary logistic regression was computed to analyze the effect of each variable on the outcome variable. During bivariate logistic regression, independent variables with a P _value of <0.25 were considered candidates for the final logistic regression model. Then all candidate variables were subjected to multiple regression analysis to identify factors significantly associated with indoor air pollution practices after adjusting for possible confounding variables in the finally fitted regression model. Hosmer and Lemeshow test was performed to evaluate the model goodness of fit by comparing the disparities between observed and predicted values. The diagnosis results revealed that the model fit the data adequately and there was no significant difference between observed and expected values (P-value = 0.44). The variance inflation factors (VIF) were used to assess if

there were multicollinearity among covariates in the fitted model. The results of diagnosis showed that none of the covariates showed the VIF value above five which shows the absence of collinearity among covariates. The strength of association was expressed in an adjusted odds ratio with 95% confidence interval and P -value <0.05 was used as cutoff points to declare the statistical significance in the final model.

Ethical considerations

Ethical approval and clearance were obtained from the Adama Hospital Medical College, Institutional Ethical Review Committee. The health and administrative offices of the town received a letter of cooperation and ethical approval. Then, approval was obtained by contacting officials at various levels. The college institutional review board approved the information sheet and consent form, which was then attached to the questionnaire's first page. Before beginning to collect data, data collectors read each information sheet and consent form to enable mothers to make the right decisions. Furthermore, all the study participants were informed about the study's objective, benefits, and risks. Verbal consent of all study subjects was obtained before data collection. Participants were also informed that they have the full right to discontinue or refuse to participate in the study. To ensure confidentiality, the name of the interviewee wasn't written on the questionnaire. Each respondent was assured that the information provided by them was confidential and used only for research. Moreover, no risk or harm was anticipated in the participation of the study.

Results

Socio-demographic characteristics of respondents

A total of 422 mothers were interviewed with a response rate of 99.52%. The median age was 37 years with an interquartile range of 16 years. The minimum and maximum age was 18 and 82 years respectively. One hundred forty-three (34.2%) of them founded in the age category of 31 – 40 years. One hundred eighty-five (44.3%) households had under five years children. Among the respondents, 284 (67.9%) were Oromo by ethnicity and 180 (43.1%) were Orthodox in religion. Regarding educational status, 105 (25.1%) had elementary (grade 1-8) levels and 192 (45.9%) were housewives in occupation. Regarding household monthly income, 179 (42.8%) got less than 36 US$ per month (Table 1).

Table 1: Socio-demographic Characteristics among households in Olanchitiy town, East Shewa zone, Oromia, Ethiopia, 2023

**Variables (n=418)**	**Number (%)**
Family size	
< 5	284 (67.9)
≥ 5	134 (32.1)
Having under-five children	
Yes	185 (44.3)
No	233 (55.7)
Age of respondents	
18 - 30	133 (31.8)
31 -40	143 (34.2)
41 -50	89 (21.3)
51 and above	53 (12.7)
Ethnicity	
Oromo	284 (67.9)
Amhara	118 (28.2)
Gurage	14 (3.3)
Others*	2 (0.5)
Religion	
Muslim	153 (36.6)
Orthodox	180 (43.1)
Protestant	80 (19.1)
Waqefata	5 (1.2)
Educational status	
Unable to read and write	104 (24.9)
Read and write	71 (17.0)
Grade 1-8	105 (25.1)
Grade 9 -12	91 (21.8)
Diploma and above	47 (11.2)
**Occupation**	
Housewife	192 (45.9)
Merchant	94 (22.5)
Governmental employee	33 (7.9)
Daily laborer	31 (7.4)

Private employee	39 (9.3)
Farmer	29 (6.9)
Household monthly income (ETB)	
<2000	179 (42.8)
2001 – 4000	161 (38.5)
4001 - 5000	37 (8.9)
5001 and above	41 (9.8)

*=Tigire and Kembata

Housing conditions-related factors of households

Two hundred ninety-nine (71.5%) households had a separate kitchen from the main living room and 110 (26.3%) of household's kitchens or living rooms had windows and 122 (29.2%) of them had a chimney vent pipe for ventilation. One hundred fifty-four (36.8%) households used modern stoves for cooking and 291 (69.6%) of their roof were constructed with corrugated iron sheets (Table 2).

Table 2: Housing conditions-related factors among households in Olanchitiy town, East Shewa zone, Oromia, Ethiopia, 2023

**Variables (n=418)**	**Number (%)**
Having a Separate kitchen from the living room	
Yes	299 (71.5)
No	119 (28.5)
The kitchen or living room had a window	
Yes	110 (26.3)
No	308 (73.3)
The housing had a Chimney (vent pipe)	
Yes	122 (29.2)
No	296 (70.8)
Having a modern stove for cooking	
Yes	154 (36.8)
No	264 (63.2)
Type of modern stoves (n=154)	
Mixadi*	50 (32.5)
Electric stove	73 (47.4)

Midija (lakech)*	31 (20.1)
Type of roof construction	
Corrugated Iron sheet	291 (69.6)
Traditional thatch roof	125 (29.6)
Others (plastic)	2 (0.5)
Room cleanliness (dust)	
Cleaned	248 (59.3)
Not cleaned	170 (40.7)

*=Ethiopian locally-made modern stoves

Household's Sources of energy

Among the studied households, Only 131 (31.3%) were using electricity for cooking. However, most (99.3%) households used wood or charcoal for food preparation. Similarly, almost all (99%) households were using mixed sources of energy such as electricity, wood, charcoal, animal dung, and kerosene (Table 3)

Table 3: Household's sources of energy for cooking in Olanchitiy town, East Shewa zone, Oromia, Ethiopia, 2023

**Variables (n=418)**	**Number (%)**
Electricity	
Yes	131 (31.3)
No	287 (68.7)
Wood	
Yes	415 (99.3)
No	3 (0.7)
Charcoal	
Yes	415 (99.3)
No	3 (0.7)
Dung	
Yes	242 (57.9)
No	176 (42.1)
Kerosene	
Yes	35 (8.4)
No	383 (91.6)

Mixed sources utilization	
Yes	414 (99.0)
No	4 (1.0)

Household's behavioral factors

Only 19 (4.5%) of household members practiced indoor cigarette smoking. Two hundred ninety-two (69.9%) households opened their window or door while cooking. Around nine-tenths, (90.2%) of families did outside complete combustion of charcoal before entering the house. Only 69 (16.5%) households separated under five children while cooking (at least 2m from the burning fire). Most mothers (90.9%) continuously cooked in the kitchen for more than one hour without going outside the kitchen to take fresh air. Only a few mothers (2.6%) got training or follow-up on IAP from the health or energy bureau during the last six months. Forty-four households (10.5%) reported diseases during the last two weeks. Among the reported diseases, respiratory diseases accounted for 27 (61.4%) (Table 4).

Table 4: Household's behavioral factors on IAP in Olanchitiy town, East Shewa zone, Oromia, Ethiopia, 2023

**Variables (n=418)**	**Number (%)**
Indoor Cigarette smoking among households	
Yes	19 (4.5)
No	399 (95.5)
Opening window or door while cooking	
Yes	292 (69.9)
No	126 (30.1)
Outside Complete combustion of charcoal before entering the house	
Yes	377 (90.2)
No	41 (9.8)
Separation of under-five children while cooking	
Yes	69 (16.5)
No	349 (83.5)
Cooking in the kitchen for more than 1hrs without going out	
Yes	380 (90.9)
No	38 (9.1)

Training or follow-up was given on IAP by the health or energy bureau (6 months)	
Yes	11 (2.6)
No	407 (97.4)
Diseases reported among HH during the last two weeks	
Yes	44 (10.5)
No	418 (89.5)
Type of diseases reported	
Respiratory diseases	27 (61.4)
Eye diseases	13 (29.5)
Cancer	4 (9.1)

Knowledge of Indoor Air Pollution

Most of the mothers, 386 (92.3%) and 406 (97.1%) mentioned indoor air pollution as a cause of respiratory diseases and cataracts or eye problems respectively. Some mothers also mentioned indoor air pollution as a cause of some health and related problems: lung cancer 262 (62.7%), low birth weight 220 (52.6%), impaired cognitive development 214 (51.2%), and global climate change 288 (68.9%). Concerning the overall knowledge of mothers, 279 (66.7%) had good knowledge of the impact of indoor air pollution (Table 5).

Table 5: Knowledge of IAP among mothers in Olanchitiy town, East Shewa zone, Oromia, Ethiopia, 2023

**Variables (n=418)**	**Number (%)**
IAP causes respiratory diseases	
Yes	386 (92.3)
No	32 (7.7)
IAP causes lung cancer	
Yes	262 (62.7)
No	156 (37.3)
IAP causes low birth weight	
Yes	220 (52.6)
No	198 (47.4)
IAP Impaired cognitive development	
Yes	214 (51.2)

No	204 (48.8)
IAP causes cataract	
Yes	406 (97.1)
No	12 (2.9)
IAP causes global climate change	
Yes	288 (68.9)
No	130 (31.1)
Overall knowledge	
Good Knowledge	279 (66.7)
Poor knowledge	139 (33.3)

Household's indoor air pollution prevention practices

The overall good practices of households towards indoor air pollution were 188 (45.0%) with 95%CI (40.2 – 51.0) (Table 6)

Table 6: Household's IAP prevention practices in Olanchitiy town, East Shewa zone, Oromia, Ethiopia, 2023

**Variables (n=418)**	**Number (%)**
Use of separate kitchen	
Yes	299 (71.5)
No	119 (28.5)
Use of modern stove	
Yes	154 (36.8)
No	264 (63.2)
Opening window or door while cooking	
Yes	292 (69.9)
No	126 (30.1)
Separating under five children while cooking (at least 2m away from burning fires)	
Yes	69 (16.5)
No	349 (83.5)
Use of clean energy source (electricity)	
Yes	131 (31.3)
No	287 (68.7)

Outside complete combustion of charcoal	
Yes	377 (90.2)
No	41 (9.8)
Overall practice	
Good Practices	188 (45.0)
Poor Practices	230 (55.0)

Factors affecting household's indoor air pollution prevention practices

In the binary logistic regression variables such as family size, under-five children, age of respondents, educational status, occupation, household monthly income, and training were statistically significant at p-value < 0.25 and transferred to the multivariable analysis. However, after adjusting for possible confounding factors, only under-five children's and educational status were associated with indoor air pollution prevention practices at p-value ≤ 0.05.

Among households who had under-five children 48 % (AOR =0.52, 95%CI (0.33-0.82) were less likely to practice indoor air pollution prevention practices than those who didn't have under-five children. Similarly, mothers in grade 9 – 12 educational level 49% (AOR=0.51, 95%CI (0.28 - 0.92)) and diploma and above were 72% (AOR=0.28, 95%CI (0.09 - 0.84)) less likely to practice indoor air pollution prevention practices than those unable to read and write (Table 7).

Table 7: Factors affecting household IAP prevention practices in Olanchitiy town, East Shewa zone, Oromia, Ethiopia, 2023

**Variables**	**IAP prevention practices**		**COR (95%CI)**	**AOR (95%CI)**	**P - value**
	**Good N (%)**	**Poor N (%)**			
Family size					
< 5	111(39.1)	173(60.9)	2.10 (1.38 -3.19)	1.37(0.83-2.26)	0.20
≥ 5	77(57.5)	57(42.5)	1.0	1.0	
Under five children					
Yes	106(57.3)	79(42.7)	0.40(0.27 – 0.60)	**0.52(0.33-0.82)**	0.01
No	82(35.2)	151(64.8)	1.0	1.0	

Age of respondents					
18 - 30	49(36.8)	84(63.2)	0.95 (0.49 -1.85)	1.18(0.54-2.59)	0.67
31 -40	73(51.0)	70(49.0)	0.53 (0.28 – 1.02)	0.64(0.30-1.37)	0.25
41 -50	47(52.8)	42(47.2)	0.49 (0.24 – 1.00)	0.56(0.26-1.22)	0.15
51 and above	19(35.8)	34(64.2)	1.0	1.0	
Educational status					
Illiterate	36(34.6)	68(65.4)	1.0	1.0	
Read and write	27(38.0)	44(62.0)	0.86 (0.46 -1.61)	0.71 (0.36- 1.40)	0.33
Grade 1-8	43(41.0)	62(59.0)	0.76 (0.43-1.33)	0.73 (0.40 - 1.34)	0.32
Grade 9 -12	48(52.7)	43(47.3)	0.47 (0.26 -0.84)	**0.45 (0.24 - 0.88)**	**0.01**
Diploma and above	34(72.3)	13(27.7)	0.20 (0.09 -0.43)	**0.28 (0.09 -0.84)**	**0.02**
Occupation					
Housewife	82(42.7)	110(57.3)	0.51 (0.21 - 1.21)	0.56(0.23-1.37)	0.20
Merchant	50(53.2)	44(46.8)	0.33 (0.13 -0.83)	0.49(0.18-1.28)	0.14
Governmental employee	26(78.8)	7(21.2)	0.10 (0.03 – 1.32)	0.42(0.09-1.78)	0.24
Daily laborer	7(22.6)	24(77.4)	1.30 (0.40 - 4.21)	1.37(0.40-4.70)	0.61
Private employee	15(38.5)	24(61.5)	0.61 (0.21 – 1.72)	1.01(0.33-3.08)	0.98
Farmer	8(27.6)	21(72.4)	1.0	1.0	
Household monthly income (ETB)					
<2000	67(37.4)	112(62.6)	1.0	1.0	
2001 – 4000	74(46.0)	87(54.0)	0.70 (0.45- 1.08)	0.82 (0.51-1.33)	0.43
4001 - 5000	17(45.9)	20(54.1)	0.70 (0.34 -1.43)	1.14 (0.51-2.57)	0.73
5001 and above	30(73.2)	11(26.8)	0.21 (0.10 -0.46)	0.48 (0.19 – 1.17)	0.11
Training					
Yes	7(63.6)	4(36.4)	0.45 (0.13 – 1.58)	0.48(0.11-1.97)	0.31
No	181(44.5)	226(55.5)	1.0	1.0	

Abbreviations: COR crude odds ratio, AOR adjusted odds ratio, CI confidence interval, 1.0 reference category

Discussions

The study assessed the level of indoor air pollution prevention practices and associated factors among households in Olenchiti town, East Shewa zone, Oromia, Ethiopia. Based on the main findings, less than half of the households

practiced good prevention of indoor air pollution, (45.0%, 95%CI (40.2 – 51.0)). The finding was similar to a study done in a rural village in South Africa, 51% [17] and in Northwest Ethiopia, (46%) [22], however, higher than the study done in rural Jimma, (39%) [23], the difference may be due to the study time variations and methods.

Regarding household sources of energy, the majority of (68.7%) households used biomass fuel for cooking. The finding was almost similar to a World Bank report, in Africa, over half a billion people still rely on biomass fuel for cooking and heating [6]. This finding was much behind the target of the Sustainable Development Goal on Energy (SDG 7).which says to ensure universal access to clean fuel and technologies.

Concerning the housing condition of households, (28.5%) households had no separate kitchen from the main living room and cooked inside their living houses. The finding was similar to a study done in a rural town in Kenya (37%) [20], however, the finding was higher than the study done in a study conducted in rural Town of Uganda (11.5%) [21], with a slight difference may be due to study time variation and methods. Similarly. Only (36.8%) of households used improved or modern stoves for cooking, almost similar to a study done in a rural village in South Africa (27%) [17]. Regarding household behavioral factors, (69.9%) of households opened windows or doors while cooking. Similar to a study done in a rural village in South Africa (69%) [17], the possible reasons may be the knowledge of mothers regarding the impact of indoor air pollution on the household members should be increased.

Households that had under-five children were 51 % less likely to practice indoor air pollution prevention practices than those who didn’t have under-five children. Many findings suggested that mothers with under five children are so busy, much of their time is wasted on child caring, cooking, family guidance, and income generation. Similarly, mothers in grades 9 – 12 educational level (49%) and diploma and above (72%) were less likely to practice indoor air pollution prevention practices than those unable to read and write. The finding was contrary to studies done in China [26] and Bangladesh [27] which suggested that households with highly educated adults practice IAP prevention more likely than the least-educated households. The difference may be in the study area, about IAP topics not included in their high school and college curriculum and attention was not given to high school and college students.

Limitations of the Study

The study findings included household mothers from a single town. Therefore, the research results are limited to this particular town and cannot be generalized to other towns in Ethiopia.

Conclusions and recommendations

The overall good practices of households towards indoor air pollution were low compared to different findings in Ethiopia as well as in Africa. Under-five children and educational status were significantly associated with indoor air pollution prevention practices in the final model. Therefore, this study recommends the following actions to alleviate the problems of indoor air pollution in Welenchiti town. The town health office should be strengthened family planning services for households to regulate their family size. Similarly, the town educational offices should be incorporated indoor air pollution topics into regular high school and college curriculums. Further research is needed for measuring the concentration level of indoor air pollutants.

Acknowledgments

We hugely acknowledge the different levels of administrative hierarchy. Furthermore, the authors like to acknowledge the study participants, data collectors, and colleagues for their genuine cooperation during the course of the study.

References

---

1 Fullerton DG, Semple S, Kalambo F, Suseno A, Malamba R, Henderson G,et al. Biomass fuel use and indoor air pollution in homes in Malawi. Occup Environ Med, 2009

2 Langbein J. Firewood, smoke and respiratory diseases in developing countries: the neglected role of outdoor cooking, 2017, https://www.ncbi.nlm.nih.

3 Ferguson, et al. Exposure to indoor air pollution across socio-economic groups in high income countries: A scoping review of the literature and a modelling methodology, Environment International, 2020, https://doi.org/10.1016/j.envint.2020.105748

4 Barnes et al, A behavioral intervention to reduce child exposure to indoor air pollution: identifying possible target behaviors. Health Education and Behavior, in press, 2004

5 IEA. World energy outlook. Paris: International Energy Agency (IEA); 2012.

https://www.iea.org/topics/world-energy-outlook,

6 Ekouevi K, Tuntivate V. Household energy access for cooking and heating: lessons learned and the way forward: World Bank publications; 2012. https://doi.org/10.1596/978-0-8213-9604-9

7 Worku Tefera et al, Indoor and Outdoor Air Pollution- related Health Problem in Ethiopia: Review of Related Literature, Article in Ethiopian Journal of Health Development, 2016 https://www.researchgate.net/publication/313083044

8 Lim SS, Vos T, Flaxman AD, Danaei G, Shibuya K, et al. A comparative risk assessment of burden of disease and injury attributable to risk factors and risk factor clusters in 21 regions: a systematic analysis for the Global Burden of Disease Study, 2012.

9 Women's Commission for Refugee Women and Children Beyond firewood: Fuel alternatives and protection strategies for displaced women and girls, New York, 2006.

10 UNEP Near-term climate protection and clean air benefits: actions for controlling short lived climate forcers. A UNEP synthesis report. Nairobi: UNEP 2011.

11 Shen M, Chapman RS, Vermeulen R, Tian L, Zheng T, et al. Coal use, stove improvement, and adult pneumonia mortality in Xuanwei, China: a retrospective cohort study, 2009.

12 Chapman RS, He X, Blair AE, Lam Q, Improvement in household stoves and risk of chronic obstructive pulmonary disease in Xuanwei, China: retrospective cohort study. BMJ, 2005.

13 Lan Q, Chapman RS, Schreinemachers DM, Tian L, He X, Household stove improvement and risk of lung cancer in Xuanwei, China, 2002.

14 Pope DP, Mishra VK, Thompson L, Siddiqui AR, Rehfuess E, et al. Risk of low birth weight and stillbirth associated with indoor air ollution from solid fuel use in developing countries, 2010

15 Dix-Cooper L, Eskenazi B, Romero C, Balmes J, Smith KR, Neurodevelopmental performance among school age children in rural Guatemala is associated with prenatal and postnatal exposure to carbon monoxide, a marker for exposure to woodsmoke, 2012

16 Pokhrel AK, Smith KR, Khalakdina A, Deuja A, Bates MN, Case-control study of indoor cooking smoke exposure and cataract in Nepal and India, 2005

17 B. R. Barnes et al. Testing selected behaviors to reduce indoor air pollution exposure in young children in a rural village of South Africa, Health Education Research Vol.19 no.5, Oxford University Press 2004

18 Esong et al. Distribution of sources of household air pollution: a cross-sectional study in Cameroon, BMC Public Health (2021) 21:318 https://doi.org/10.1186/s12889-021-103506

19 Dida et al. Factors predisposing women and children to indoor air pollution in rural villages, Western Kenya, Archives of Public Health BMC, (2022) 80:46. https://doi.org/10.1186/s13690-022-00791-9

20 Wilkister Nyaora Moturi, Risk factors for indoor air pollution in rural households in Mauche division, Molo district, Kenya, African Health Sciences Vol 10 No 3 September 2010

21 Serunjogi, D.T. and Musoke, D. Risk Factors for Indoor Air Pollution Exposure in Households in Kasangati Town Council, Wakiso District, Uganda, African Journal of Environmental Health Sciences, Volume 7, November, 2020 http://www.ehsanonline.org

22 Adane et al. Biomass-fuelled improved cook stove intervention to prevent household air pollution in Northwest Ethiopia: a cluster randomized controlled trial, Environmental Health and Preventive Medicine (2021), https://doi.org/10.1186/s12199-020-00923

23 Faris K. Survey of Indoor Air Pollution Problems in the Rural Communities of Jimma, Southwest Ethiopia. Ethiop J Health Sci. 2002; 12(1):1–8.

24 Balidemaj, F.; Isaxon, C.; Abera, A.; Malmqvist, E. Indoor Air Pollution Exposure of Women in Adama, Ethiopia, and Assessment of Disease Burden Attributable to Risk Factor. Int. J. Environ. Res. Public Health 2021, 18, 9859. https://doi.org/10.3390/ ijerph18189859

25 K. R. Smith and S. Mehta, "The burden of disease from indoor air pollution in developing countries: comparison of estimates," International Journal of Hygiene and Environmental Health, vol. 206, no. 4-5, pp. 279–289, 2003

26 Maya Papineau et al, Distributional determinants of household air pollution in China, Cambridge University Press, 2009. https://www.cambridge.org/core,

27 Susmita et al, who suffers from indoor air pollution? Evidence from Bangladesh, Published by Oxford University Press in association with The London School of Hygiene and Tropical Medicine, 2006.

28 Audrey Le et al. Perception and Barriers to Indoor Air Quality and Perceived Impact on Respiratory Health: An Assessment in Rural Honduras, Hindawi Publishing Corporation Advances in Public Health Volume 2014, http://dx.doi.org/10.1155/2014/105260

29 Dida et al. Factors predisposing women and children to indoor air pollution in rural villages, Western Kenya, Archives of Public Health BMC, 2022, 80:46. https://doi.org/10.1186/s13690-022-00791-9

30 E. Coker, et al. Household air pollution profiles associated with persistent childhood cough in urban Uganda, Environment International, 2020, https://doi.org/10.1016/j.envint.2020.105471

31 Dyjack D, Soret S, Chen L, Hwang R, Nazari N, Gaede D. Re sidential environmental risks for reproductive age women in developing countries. J Midwifery Womens Health, 2005

32 Central Statistical Authority (CSA) Ethiopia. Statistical Abstract, Addis Ababa, Ethiopia, 2015

33 Girsha W , Adlo A, Garoma D, Beggi S. Assessment of Water, Sanitation and Hygiene Status of Households in Welenchiti Town, Boset Woreda, East Shoa Zone, Ethiopia. Science Journal of Public Health, 2016 doi: 10.11648/j.sjph.20160406.13

Supporting information

S1 File. (PDF)

S1 Data Set. (SAV)

Date: August 01, 2023

Srijan Lal Shrestha, Ph.D.
Academic Editor
PLOS ONE

Subject: Response to Reviewers

First of all, thank you all, for your nice comments and suggestions, we try to correct the comments as the following:

1. We tried to edit our manuscript according to PLOS ONE's style requirements
2. Rephrased and cited overlapping text with the previous publication
3. Ethical statements are clarified in the document
4. The funding is more clarified as below:

Worku Dugassa Girsha wined the grant for the research. This study was entirely funded by the Oromia Regional Health Bureau. The total grant for the project was 156,000 Ethiopian birr (equivalent to 2,600 US$). The Oromia Regional Health Bureau funded only the indicated financial support, whereas, our institution, Adama hospital medical college provided vehicles with fuel for supervision. All authors are permanent workers of Adama hospital medical college and received regular monthly salaries, but not related to this study. Website of Oromia Regional Health Bureau: Email: ohbhead@ethionet.et. The funders had no role in study design, data collection, and analysis, decision to publish, or preparation of the manuscript.

5. The following Supporting information is provided
   a. S1 File. (Information sheet, consent form, and English version questionnaires (PDF)
   b. S1 Data (SPSS Data Set) (SAV)
6. For systematic sampling, sampling frame or list and Stratification statistics were clarified
7. For logistic regression, basic assumptions (normality, model fitness, and multicollinearity) were checked and revised (included in the manuscript)
8. Authors' contributions corrected according to guideline contents
9. A marked-up copy of our manuscript that highlighted changes with yellow color and an unmarked version of our revised paper without color changes are attached.
10. Laboratory protocols are not used or applied in the study and we are free of that.

With all the best regards

Worku Dugassa Girsha
