# Supplementary material for: Indoor air pollution prevention practices and associated factors among household mothers in Olenchiti town, Oromia, Ethiopia
Source: PLoS One. 2024 Jan 19;19(1):e0296706. doi: 10.1371/journal.pone.0296706 (PMC10798486; doi:10.1371/journal.pone.0296706)
Supplement: Attachment — Submitted filename: A letter Response to Reviewers.docx [file pone.0296706.s003.docx]

Date: August 01, 2023

Srijan Lal Shrestha, Ph.D.

Academic Editor

PLOS ONE

**Subject: Response to Reviewers**

First of all, thank you all, for your nice comments and suggestions, we try to correct the comments as the following:

1. We tried to edit our manuscript according to PLOS ONE's style requirements
2. Rephrased and cited overlapping text with the previous publication
3. Ethical statements are clarified in the document
4. The funding is more clarified as below:

Worku Dugassa Girsha wined the grant for the research. This study was entirely funded by the Oromia Regional Health Bureau. The total grant for the project was 156,000 Ethiopian birr (equivalent to 2,600 US$). The Oromia Regional Health Bureau funded only the indicated financial support, whereas, our institution, Adama hospital medical college provided vehicles with fuel for supervision. All authors are permanent workers of Adama hospital medical college and received regular monthly salaries, but not related to this study. Website of Oromia Regional Health Bureau: Email: [ohbhead@ethionet.et](mailto:ohbhead@ethionet.et). The funders had no role in study design, data collection, and analysis, decision to publish, or preparation of the manuscript.

5. The following Supporting information is provided
   a. S1 File. (Information sheet, consent form, and English version questionnaires (PDF)
   b. S1 Data (SPSS Data Set) (SAV)
6. For systematic sampling, sampling frame or list and Stratification statistics were clarified
7. For logistic regression, basic assumptions (normality, model fitness, and multicollinearity) were checked and revised (included in the manuscript)
8. Authors' contributions corrected according to guideline contents
9. A marked-up copy of our manuscript that highlighted changes with yellow color and an unmarked version of our revised paper without color changes are attached.
10. Laboratory protocols are not used or applied in the study and we are free of that.

With all the best regards

Worku Dugassa Girsha
