# Supplementary material for: Indoor air pollution prevention practices and associated factors among household mothers in Olenchiti town, Oromia, Ethiopia
Source: PLoS One. 2024 Jan 19;19(1):e0296706. doi: 10.1371/journal.pone.0296706 (PMC10798486; doi:10.1371/journal.pone.0296706)
Supplement: Attachment — Submitted filename: Response to Reviewers.docx [file pone.0296706.s005.docx]

Date: October 20, 2023

To Academic Editors and Reviewers

PLOS ONE

**Subject: Response to Reviewers**

First of all, thank you all, for your nice comments and suggestions, many thanks for your genuine support and many help. We tried to correct the comments as the following:

**Reviewer #1**

1. Line 115-16: The normality test was corrected with the Kolmogorov-Smirnov test
2. Line 117-18: A higher p-value cut-off of 0.25 was used to transfer independent variables into the multivariable analysis, so as not to miss important variables in the final model (Reference 34) and we removed them from the result part.
3. Line 117-19: Concerning sampling weight, the town has two kebeles (strata) and we did proportional allocations for each kebele (Kebele one had a proportional allocation of 304 mothers, and Kebele two had a proportional allocation of 116 mothers) (line 86 -87). In addition, in our study, the respondents or interviewers are only mothers among households.
4. Line 119: Multiple regression analysis was changed with a multivariate logistic regression model
5. Line 123-24: VIF values were reported separately in Table 7 for each variable. The variance inflation factor greater than five was used as an indicator of multicollinearity (Reference 35)

**Reviewer #2**

1. Line 115: Corrected like this, the normality of the age variable was checked with the Kolmogorov-Smirnov Test (P<0.01) and the data was not normally distributed.
2. Line 23: The sentence was corrected according to the given comments, the overall good practices of mothers towards the prevention of indoor air pollution was 188 (45.0%).
3. Line 80: Corrected with, Z value at 95% confidence interval =1.96
4. Line 87/88: The sampling interval was 19 (the total population of 8028 divided by a sample size of 420), then, the selected households were contacted for data collection. Since we had a sampling frame (lists) we did it easily on the paper, and then we identified and contacted for data collection.
5. Line 121: The value of the Hosmer and Lemeshow test was (P-value = 0.44)
6. Line 123: The value of VIF corrected like this, the variance inflation factor greater than five was used as an indicator of multicollinearity. All the covariate values of VIF were less than one point five (VIF-value < 1.5), which showed the absence of collinearity among covariates. VIF values were reported separately in Table 7 for each variable.
7. Line 142: Yes, in reality, the knowledge may vary with a huge range of age intervals, however, didn’t show significance with the outcome variable, which may be because of data quality
8. Line 184: Indoor air pollution prevention practices were categorized into good practice or poor practice by considering the percentage of mothers who correctly answered or fulfilled the given six questionnaires such as the use of a separate kitchen, opening a window while cooking, using of a modern stove, outside complete combustion of charcoal, separating under five children while cooking, and use of clean energy sources (Line 110-113).
9. Line 197: AOR for non-significant variables used to show more clarity, even though, some variables are transferred to the final model, didn’t indicate significance, therefore, the values are indicated for clarity.

With all the best regards

Worku Dugassa Girsha

Thanks again for your genuine support!
