# Supplementary material for: Indoor air pollution prevention practices and associated factors among household mothers in Olenchiti town, Oromia, Ethiopia
Source: PLoS One. 2024 Jan 19;19(1):e0296706. doi: 10.1371/journal.pone.0296706 (PMC10798486; doi:10.1371/journal.pone.0296706)
Supplement: Attachment — Submitted filename: Response to Reviewers.docx [file pone.0296706.s006.docx]

**Date: November 23, 2023**

**To Academic Editors and Reviewers**

**PLOS ONE**

**Subject: Response to Reviewers**

Again, thank you all, for your good comments and suggestions, many thanks for your support and many help. We tried to correct the comments as the following:

**Response to Academic Editor (Journal Requirement):**

1. In the previous reviewers comments, two references were asked to make it clear for the cut-off p-value to transfer variables to the final model (reference 34, line 118) and the cut-off points for VIF (reference 35, line 124); therefore, the two new references were added previously for clarity.

**Responses to reviewer one**

2. Comment in lines 84–91, concerning sampling weight, different references indicated that sampling weights are the number of individuals in the population each respondent in the sample is representing. Accordingly, in our study, the respondents, or interviewers, are only mothers in households. However, only three households had no the required mother, and the next household was substituted. In addition, only two households had mothers with equal family representatives, selected by the lottery method. The detailed work was done in lines 84–91.
3. Comment in lines 125–126: Concerning the Variance Inflation Factor (VIF) cut-off point for logistic regression analysis, yes, we have seen different book references (evidences). Most research papers consider a VIF > 10 as an indicator of multicollinearity, but some choose a more conservative threshold of 5 or even 2.5. VIF > 5 is a cause for concern, and VIF > 10 indicates a serious collinearity problem. So that, we chose the more average conservative greater than five. The following references are attached:

| **VIF Threshold** | **Reference Type** | **Reference** |
| --- | --- | --- |
| VIF > 10 is problematic | Book | Vittinghoff E. Regression Methods in Biostatistics: Linear, Logistic, Survival, and Repeated Measures Models. Springer; 2005. |
| VIF > 5 or VIF > 10 is problematic | Book | ames G, Witten D, Hastie T, Tibshirani R. An Introduction to Statistical Learning: With Applications in R. 1st ed. 2013, Corr. 7th printing 2017 edition. |
| VIF > 5 is cause for concern and VIF > 10 indicates a serious collinearity problem | Book | Menard S. Applied Logistic Regression Analysis. 2nd edition. SAGE Publications, Inc; 2001. |
| VIF ≥ 2.5 indicates considerable collinearity | Research Paper | Johnston R, Jones K, Manley D. Confounding and collinearity in regression analysis: a cautionary tale and an alternative procedure, illustrated by studies of British voting behaviour. Qual Quant. 2018;52(4):1957-1976. doi:10.1007/s11135-017-0584-6, 2018 |

4. Comment in line 126-128: Thanks, The variance inflation factors (VIF) result was taken under the regression result part (Lines 91–93).
5. Comment on Table 7. Thank you very much. We completely revised the regression table, and the overall p-value based on the Wald test to transfer candidates to the final model was revised according to your comments. Upon the revised value, the overall p-value for the age category (P<0.42) was greater than P<0.25, therefore the age category was omitted from the final model. Then the AOR with the P-value for each variable in the final model was rechecked and corrected. Based on revision, the educational level of diploma and above was not statistically significant (p<0.06). Furthermore, the result, discussion, conclusion, and recommendations were edited accordingly. Thank you once again for your professional input.

With all the best regards

Worku Dugassa Girsha

Thanks again for your support!
